# Pathogen-associated molecular patterns alter molecular clock gene expression in mouse splenocytes

**DOI:** 10.1371/journal.pone.0189949

**Published:** 2017-12-18

**Authors:** Adam C. Silver

**Affiliations:** Department of Biology, University of Hartford, West Hartford, CT, United States; University of Lübeck, GERMANY

## Abstract

Circadian rhythms are endogenous 24-h oscillations that influence a multitude of physiological processes. The pathogen-associated molecular pattern (PAMP), lipopolysaccharide, has been shown to modify the circadian molecular clock. The aim of this study was to determine if other PAMPs alter clock gene expression. Therefore, mRNA levels of clock genes (*Per2*, *Bmal1*, *Rev-erbα*, and *Dbp*) were measured after an *ex vivo* challenge with several PAMPs and to further test the relevance of PAMP alteration of the molecular clock, an *in vivo* poly(I:C) challenge was performed. This study revealed that several other PAMPs are also capable of altering clock gene expression.

## Introduction

Many crucial behaviors and biological processes are regulated in a circadian-dependent manner [1]. The master clock, located within the suprachiasmatic nucleus (SCN) of the hypothalamus, endogenously generates these 24-h oscillations [2]. Synchronization of the clock is affected by many environmental cues, such as exercise or temperature, but daily changes in light intensity have the greatest influence on clock entrainment [2]. In addition to regulating nearly all aspects of physiology and behavior, the master clock also helps entrain peripheral oscillators throughout the body [3–5]. On a molecular level, circadian oscillations are generated by at least three interlocking transcriptional-translational feedback loops; however, the core is composed of the *Period* (*Per1-3*), *Cryptochrome* (*Cry1-2*), *Bmal1* (brain and muscle ARNT-like 1) and *Clock* [6,7] genes. Several other genes are involved in secondary feedback loops, such as *Rev-erbα* (*Nr1d1*) and *Dbp* (D-site albumin promoter-binding protein), which in addition to their role in regulating the molecular clock, function as transcription factors that regulate expression of clock-controlled genes (*i*.*e*., genes regulated by the clock but do not influence the clock itself) [6,7].

Alterations of circadian rhythms in the form of nightshift work or genetic abnormalities have been linked to pathologies, such as sleep disorders, depression, and increased risk of cancer [8–11]. Lipopolysaccharide (LPS), a large molecule located on the outer membrane of Gram-negative bacteria, is also capable of altering expression of molecular clock components [7,12–15]. LPS and other conserved microbial components (*e*.*g*., proteins, nucleic acids, and lipoproteins), referred to as pathogen-associated molecular patterns (PAMPs), are detected via innate immune recognition receptors such as Toll-like receptors (TLRs) [16]. To date, 13 functional TLRs have been identified (TLR1-10 in humans and TLR1-9 and TLR11-13 in mice) and each TLR detects a different PAMP. This specific TLR-PAMP interaction directs both innate and adaptive immune responses [16].

The aim of this study was to determine if PAMPs other than LPS alter expression of the molecular clock.

## Materials and methods

### Animals

Eight-week-old C57BL/6J male mice (The Jackson Laboratory) were fed rodent chow ad libitum, maintained under constant environmental conditions and entrained to a 12 h light / 12 h dark cycle (light period from 7:00 a.m. to 7:00 p.m.) for 2 weeks before experiments. For the *ex vivo* and *in vivo* challenge experiments, animals were euthanized at 8:00 a.m. or 8:00 p.m., which corresponds with Zeitgeber time (ZT) 1 and 13, respectively. To assess *Tlr* expression levels over the daily light-dark cycle, animals were sacrificed every 4 h (ZT3, ZT7, ZT11, ZT15, ZT19, and ZT23), over a 24-h period. Tissues were immediately collected for further processing as described below. During the study, animal care and treatment complied with National Institutes of Health policy, were in accordance with institutional guidelines, and were approved by the University of Hartford Animal Institutional Animal Care and Use committee.

### Splenocyte isolation

Spleens were collected in RPMI 1640 (Invitrogen Life Technologies) supplemented with 10% FBS. Each spleen was homogenized in 2 ml of medium and filtered through a 40 μM nylon cell strainer (BD Biosciences). Spleens and splenocyte suspensions were kept on ice throughout the entire procedure. Immediately after splenocytes were isolated from all of the spleens, they were challenged with various PAMPs. To assess *Tlr* expression levels over the daily light-dark cycle, approximately 5 x 10^5^ splenocytes were suspended in 300 μl of RLT buffer (Invitrogen) containing β-Mercaptoethanol and stored at -80°C until RNA extraction was performed as described below.

### *ex vivo* PAMP challenge

Approximately 5 x 10^5^ cells per well were added to 12-well culture plates (Corning) and were challenged with 1 ml of medium containing heat-killed *Listeria monocytogenes* (HKLM, 5 x 10^7^), poly(I:C) (250 ng/ml), lipopolysaccharide (LPS, from *Escherichia coli* K12, 5 μg / ml), flagellin (FLA-ST, from *Salmonella typhimurium*, 500 ng/ml), ssRNA40 (1.25 μg/ml), or ODN1826 (synthetic oligonucleotides containing unmethylated CpG motifs, 5 μM). TLR agonists were purchased from InvivoGen. After the plates were incubated at 37°C in 5% CO_2_ for 3.5 h, the supernatant was removed, and the cells were washed with PBS. 300 μl of RLT buffer (Invitrogen) containing β-Mercaptoethanol was then added to each of the wells. Cell lysates were stored at -80°C until RNA extraction was performed as described below.

### *in vivo* poly(I:C) challenge to assess influence on clock expression

Mice were injected intraperitoneally with 30 μg poly(I:C) (InvivoGen) or PBS at ZT1 and ZT13. Mice were sacrificed 3.5 or 48 h after challenge, in two separate experiments. Spleens were collected and cells isolated as described above. Approximately 10^6^ cells were pelleted and resuspended in 600 μl of RLT buffer (Invitrogen) containing β-Mercaptoethanol. Cell lysates were stored at -80°C until RNA extraction was performed as described below.

### RNA extraction and quantitative PCR

RNA from splenocytes was isolated using the RNeasy Mini kit (Qiagen) in conjunction with the RNase-Free DNase Set (Qiagen). cDNA was synthesized using the high capacity cDNA reverse transcription kit according to manufacturer’s instructions (ThermoFisher). Relative quantitation of mRNA levels was performed by quantitative PCR via TaqMan Gene Expression Assays (ThermoFisher) and TaqMan Gene Expression Master Mix (ThermoFisher) using a StepOnePlus (ThermoFisher) system. Analyses were performed using the standard curve method with β-actin as the normalizing endogenous control. Relative quantitation values were determined by dividing the relative quantity for the target gene by the relative quantity for β-actin. The following TaqMan Gene Expression Assays were used (ThermoFisher): *Actb* Mm00607939_s1, *Per2* Mm00478113_m1, *Rev-erbα* Mm00520708_m1, *Bmal1* Mm00500226_m1, *Dbp* Mm00497539_m1, *Tlr1* Mm00446095_m1, *Tlr2* Mm00442346_m1, *Tlr3* Mm01207404_m1, *Tlr5* Mm00546288_s1, *Tlr6* Mm02529782_s1.

### Statistical analysis

One-way ANOVA with the Dunnett’s posttest was used to assess differences between means of clock gene expression after PAMP challenge versus the control (PBS challenge) *in vitro* and *in vivo*. One-way ANOVA with the Dunnett’s posttest was used to assess daily variations in *Tlr* and clock gene expression between the acrophase and other time points. Two-way ANOVA with the Tukey posttest, treating time and treatment as independent factors, was used to assess differences between means of clock gene expression after PAMP challenge versus the control (PBS challenge). A two-tailed t test was used to assess differences between means of clock gene expression after PAMP challenge versus the control (PBC challenge) at 48-h post challenge. All analyses were performed using Prism 7.0a (GraphPad).

## Results

### *ex vivo* PAMP challenge

**Fig 1:**
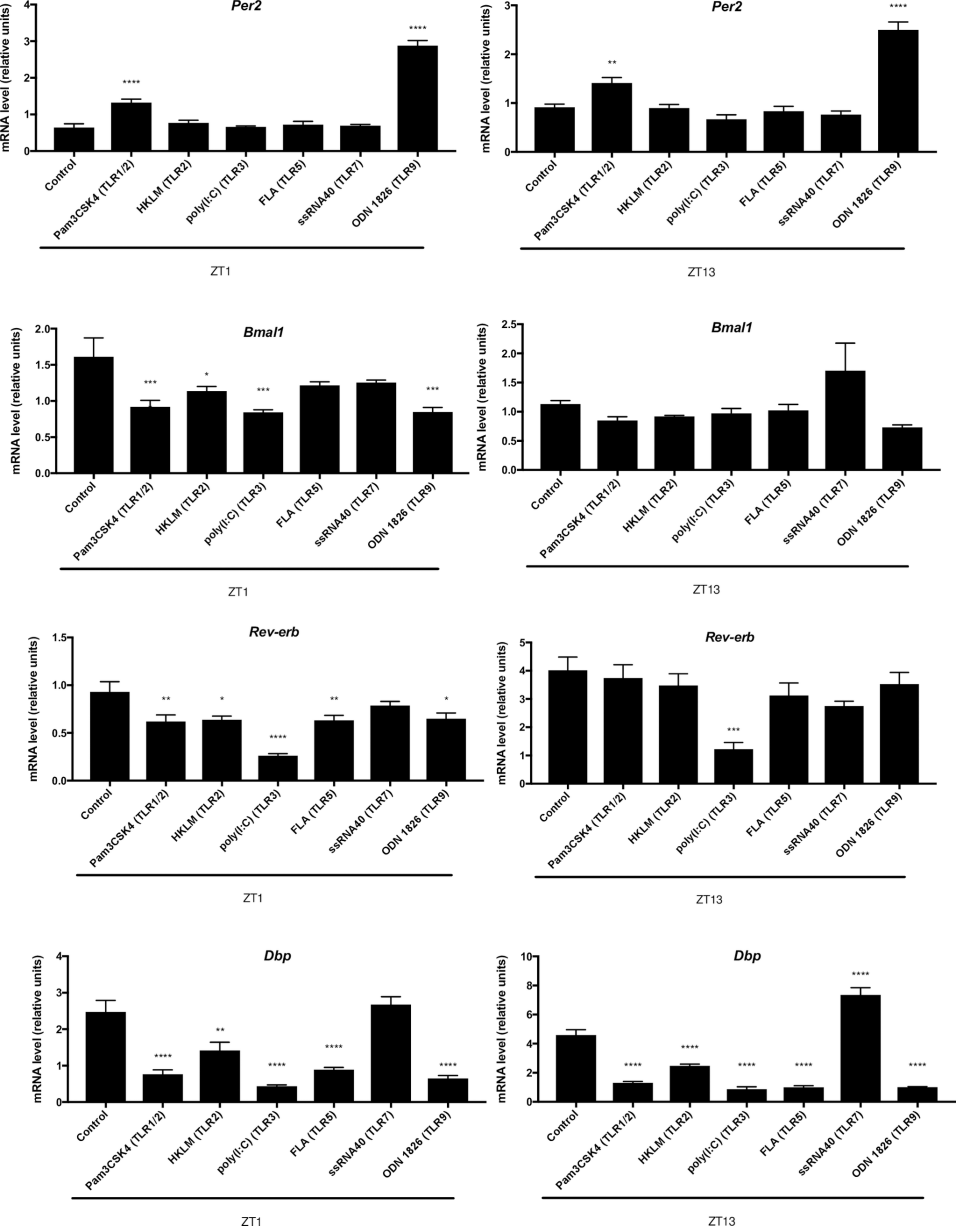
Altered clock gene expression in mouse splenocytes after PAMP challenge *ex vivo*. Splenocytes were isolated at ZT1 or ZT13, and challenged with PAMPs targeting different TLRs. Relative clock mRNA levels (normalized to β-actin) were determined by qPCR 3.5 h after challenge. Data are mean + SEM of 5 animals per time point. *p < 0.05, **p < 0.01, *** *p* < 0.001, **** *p <* 0.0001, significantly different from the control (PBS challenge) as per one-way ANOVA with the Dunnett’s posttest.

Previously, LPS, the TLR4 ligand, was shown to alter clock gene expression in various cell types and animal models [12–15,17]. In order to determine if other TLR agonists influence clock gene expression, mice were sacrificed at ZT1 or ZT13, and mouse splenocytes were challenged *ex vivo* with PAMPs targeting specific TLRs. After 3.5 h, quantitative PCR was used to assess mRNA levels of core clock genes, *Per2* and *Bmal1*, as well as ancillary genes, *Rev-erbα* and *Dbp*. After challenge with Pam3CSK4, which triggers the TLR1/2 heterodimer, expression levels of *Per2* were higher in splenocytes at ZT1 (*p*_ANOVA_ < 0.0001) and ZT13 (*p*_ANOVA_ < 0.01), while *Dbp* levels were significantly lower at both ZT1 (*p*_ANOVA_ < 0.0001; Fig 1) and ZT13 (*p*_ANOVA_ < 0.0001; Fig 1) than those in the unchallenged controls. *Bmal1* and *Rev-erbα* expression levels were lower at ZT1 (*p*_ANOVA_ < 0.001; *p*_ANOVA_ < 0.01; Fig 1) but not significantly different at ZT13 (Fig 1) after Pam3CSK4 challenge, when compared to the controls. Challenge with the TLR2 ligand, HKLM, led to lower mRNA levels of *Bmal1* at ZT1 (*p*_ANOVA_ < 0.05), *Rev-erbα* at ZT1 (*p*_ANOVA_ < 0.05), and *Dbp* at ZT1 and ZT13 (*p*_ANOVA_ < 0.01; *p*_ANOVA_ < 0.001) when compared to the controls (Fig 1). Significantly lower mRNA levels were observed for *Bmal1* at ZT1 (*p*_ANOVA_ < 0.001), *Rev-erbα* at ZT1 (*p*_ANOVA_ < 0.0001) and ZT13 (*p*_ANOVA_ < 0.001), and *Dbp* at ZT1 (*p*_ANOVA_ < 0.0001) and ZT13 (*p*_ANOVA_ < 0.0001) after challenge with the TLR3 ligand, poly(I:C), when compared to the controls (Fig 1). Challenge with the TLR5 ligand, FLA-ST, generated significantly lower amounts of *Rev-erbα* at ZT1 (*p*_ANOVA_ < 0.01) and *Dbp* at ZT1 (*p*_ANOVA_ < 0.0001) and ZT13 (*p*_ANOVA_ < 0.0001) (Fig 1). After challenge with ssRNA40, the TLR7 ligand, increased *Dbp* mRNA levels were observed at ZT13 (*p*_ANOVA_ < 0.0001; Fig 1). Lastly, after challenge with ODN 1826, a ligand that activates TLR9, mRNA levels of *Per2* were increased at ZT1 (*p*_ANOVA_ < 0.0001) and ZT13 (*p*_ANOVA_ < 0.0001), and lower mRNA levels were detected for *Bmal1* at ZT1 (*p*_ANOVA_ < 0.001), *Rev-erbα* at ZT1 (*p*_ANOVA_ < 0.05), and *Dbp* at ZT1 (*p*_ANOVA_ < 0.0001) and ZT13 (*p*_ANOVA_ < 0.0001), when compared to the unchallenged controls (Fig 1). This revealed that all of the PAMPs tested, significantly altered clock gene expression in mouse splenocytes.

### *in vivo* TLR3 ligand challenge

**Fig 2:**
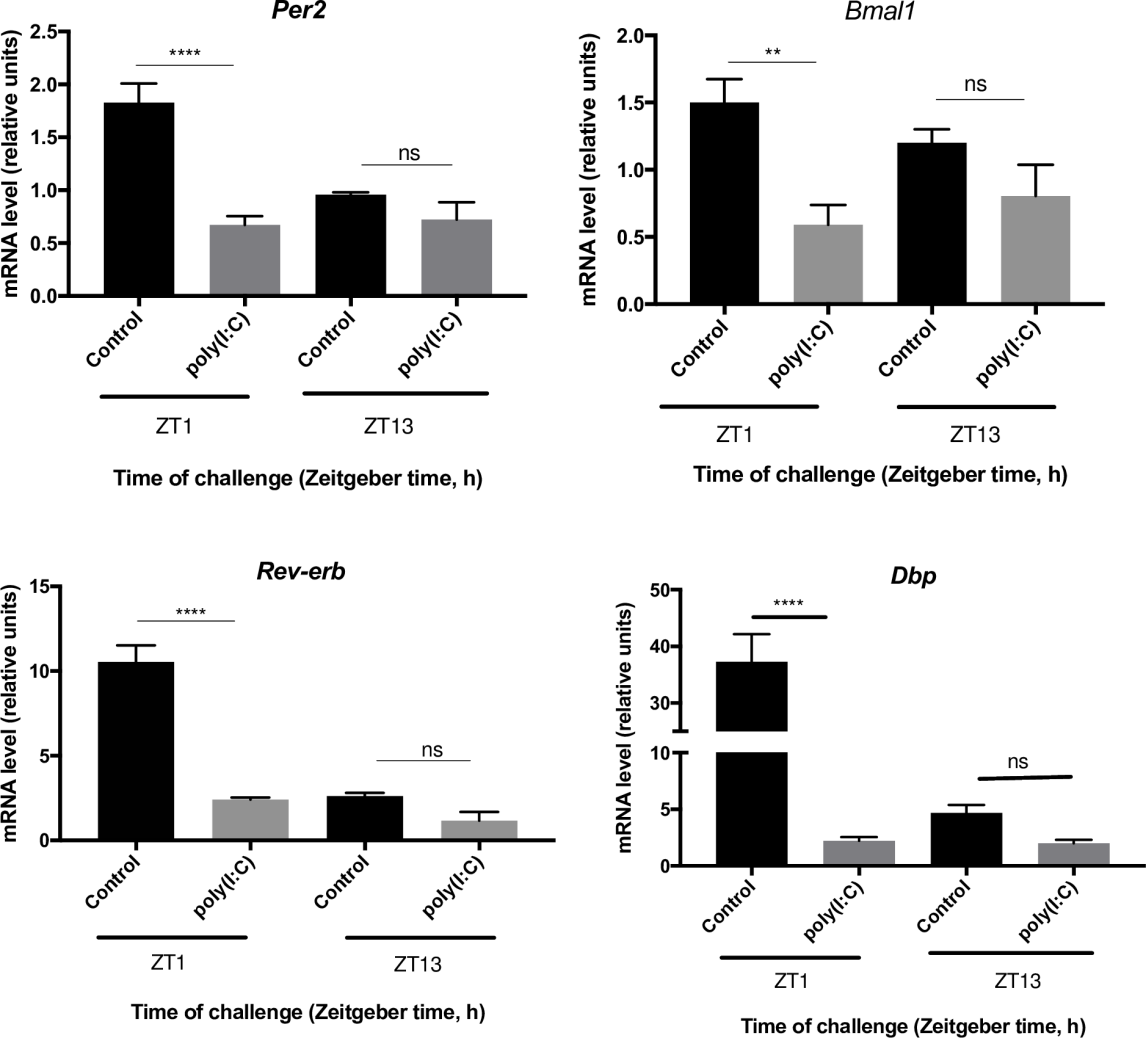
Clock gene expression after poly(I:C) challenge *in vivo*. Mice were challenged with poly(I:C) or PBS at ZT1 or ZT13 and relative clock mRNA levels (normalized to β-actin) were determined by qPCR 3.5 h after challenge in splenocytes. Data are mean + SEM of 5 animals per time point. ns, not significantly different, *p < 0.05, **p < 0.01, *** *p* < 0.001, **** *p <* 0.0001, significantly different from the control (PBS challenge) as per two-way ANOVA, with the Tukey posttest.

Next, to determine if these results could be recapitulated *in vivo* for one of the TLR agonists, mice were challenged intraperitoneally with either poly(I:C) or phosphate buffered saline (PBS) at ZT1 or ZT13. Splenocytes were isolated after 3.5 h, and clock mRNA levels were assessed via qPCR. In agreement with the *ex vivo* experiments (Fig 1), poly(I:C) challenge induced significantly lower expression levels for *Bmal1* (*p*_ANOVA_ < 0.01), *Rev-erbα* (*p*_ANOVA_ < 0.0001), and *Dbp* (*p*_ANOVA_ < 0.0001) at ZT1, while no changes were detected in *Per2* and *Bmal1* expression at ZT13, compared to the control groups (Fig 2). Some differences were observed between the *ex vivo* and *in vivo* challenge experiments, as significantly lower *Per2* amounts were detected when compared to the control at ZT1 (*p*_ANOVA_ < 0.0001; Fig 2), which was not observed *ex vivo* (Fig 1). In addition, *Rev-erbα* and *Dbp* expression was not affected when mice were challenged at ZT13 (Fig 2), but expression levels were significantly lower when challenged *ex vivo* (Fig 1). In order to verify the clock’s apparent recovery 3.5 h post-challenge at ZT13 (Fig 2), a subsequent experiment was conducted in which mice were challenged with poly(I:C) or PBS at ZT13, splenocytes were isolated after 48 h, and clock mRNA levels were assessed via qPCR. This revealed that 48-h after poly(I:C) challenge, clock gene expression remained not significantly different from the controls (S1 Fig).

## Discussion

The circadian clock regulates homeostasis from the organismal level (*e*.*g*., sleep-wake cycle) down to the molecular level (*e*.*g*., gene expression) and does so for nearly all aspects of physiology and behavior [1]. The immune system and circadian clock form an intricate overlapping web, and the circadian clock has been shown to regulate several facets of the immune system [7,18,19]. In turn, the immune response plays a role in influencing the clock. Numerous studies involving a variety of animal models (*e*.*g*., mice, rats, and human subjects) have revealed that LPS, a PAMP recognized by TLR4, alters clock gene expression in various cell types and tissues (*e*.*g*., peritoneal macrophages, suprachiasmatic nuclei, and peripheral blood leukocytes) [12–15,17,20]. In the current study, I demonstrate that, like LPS, other PAMPs can alter clock gene expression.

All of the PAMPs tested significantly altered expression of at least one clock gene in mouse splenocytes (Fig 1). *Dbp* was the gene most affected by PAMP challenge, as its expression was significantly reduced after challenge with all the PAMPs that were assessed, except for ssRNA40 at ZT13, which led to an increase in *Dbp* expression (Fig 1). Even though not all PAMPs altered expression for every gene assessed, the disruption of *Dbp*, a clock controlled transcription factor, suggests that molecular clock outputs (*i*.*e*., downstream targets of the clock) could still be significantly affected. For example, DBP has been shown to play a role in regulating locomotor activity and sleep regulation, as well as maintaining proper renal function and regulating xenobiotic metabolism [21–24].

While some PAMPs were consistent in their effect on gene expression, as they led to expression levels that were significantly different than the control, regardless of the time of challenge, several others resulted in altered expression of a particular gene at only one of the two time points assessed (Fig 1). A much more dramatic effect on clock expression was observed when splenocytes were challenged at ZT1 when compared to ZT13 (Fig 1). It has previously been shown that TLR9 is a direct target of the molecular clock and *Tlr9* expression varies throughout the day in mouse spleen, while *Tlr4* and *Tlr7* expression do not [25,26]. Here, daily variations of *Tlr1*, *3*, *and 5*, but not *Tlr2*, expression were observed in the spleen (S2 Fig). Even though *Tlr2* and *Tlr7* do not experience daily changes in gene expression, it is possible that they could exhibit daily variations in responsiveness, as daily variations in response to LPS have been observed, despite a lack of circadian oscillations in *Tlr4* expression in splenic macrophages and splenocytes [25–29]. Daily variations to LPS challenge are most likely due to daily variations of multiple components within the LPS-response pathway, rather than daily changes in *Tlr4* expression [26]. Therefore, it is possible several TLRs exhibit time-dependent differences in responsiveness, which could account for some of the time-of-challenge-dependent effects observed on the clock as demonstrated in the current study. For example, a challenge at the time of peak TLR responsiveness could lead to a greater influence on the molecular clock. In addition, clock genes have previously been shown to oscillate in the spleen [26], therefore, a PAMP that decreases expression would have a greater observable impact during time of peak clock gene expression, compared to its nadir.

Following an *in vivo* poly(I:C) challenge at ZT1, expression of *Per2*, *Bmal1*, *Rev-erbα*, and *Dbp* were all significantly lower than the controls, while significant differences in gene expression were not observed when mice were challenged at ZT13 (Fig 2). These results are consistent with the overall trend observed in the *ex vivo* challenge, which suggests the time of challenge plays a role in the degree of impact on the clock, as discussed above. When comparing the results from the *ex vivo* and *in vivo* poly(I:C) challenges, some discrepancies were observed (Figs 1 and 2). It has previously been shown that immune cell trafficking within the spleen is under circadian regulation and clock gene expression of splenocyte subtypes varies over the course of the day as well [26,30]. Therefore, the splenocytes challenged *ex vivo* are a snapshot of cells harvested at ZT1 and ZT13, whereas for the *in vivo* challenge, the spleen cell population and expression of clock genes is in flux during the 3.5 h between challenge and cell harvest. This could account for observing decreased expression levels at ZT1 for all genes (both *in vivo* and *ex vivo*) except for *Per2* in the *ex vivo* challenged, and not observing a difference at ZT13 for all genes (both *in vivo* and *ex vivo*) except for *Rev-erbα* and *Dbp*, which were significantly lower in splenocytes during the *ex vivo* challenge.

In the current study, individual PAMPs were shown to alter expression of the molecular clock. However, upon pathogen infection of a host, multiple TLRs are potentially activated by different microbial components. For example, influenza virus activates TLR3, 7, and 8 [31] and TLR2, 4, and 9 are involved in *Streptococcus pneumoniae* recognition [32–34]. Therefore, it is plausible that an infection which activates multiple TLRs could have a synergistic effect on the molecular clock. Since the circadian clock has been shown to direct various immune responses [25,26,29], clock alteration caused by TLR activation, could severely impact immune regulation. The immune dysregulation that occurs during infection (*e*.*g*., influenza virus), which to a certain extent could be mediated via clock alteration, could potentially predispose an individual to a secondary infection (*e*.*g*. *S*. *pneumoniae*) [35,36].

Here, clock expression was altered in immune cells, which directly interact with PAMPs. Further investigation is warranted to determine if these changes in clock gene expression are observed systemically, including but not limited to the SCN. One of the hallmark symptoms upon infection is the feeling of fatigue. It is intriguing to speculate that disturbances to the central clock via PAMP-TLR interaction could lead to an altered sleep state, which could be triggered in order to maximize host recovery during infection [7,37]. In addition to the production of microRNAs following TLR activation, which was shown to alter clock function [17], TLR-PAMP interaction also leads to the induction of cytokines, which in addition to their antimicrobial role, also contribute to the regulation of sleep [37] and influence clock gene expression [38]. In addition to its role in infection, the cytokine TNF has been linked to debilitating fatigue in rheumatoid arthritis patients [39], altered clock gene expression and prolonged rest periods in mice [38]. Therefore, a better understanding how infection alters the circadian clock could lead to novel therapies for both autoimmune and infectious diseases.

## Supporting information

S1 Fig Clock gene expression after poly(I:C) challenge *in vivo*. Mice were challenged with poly(I:C) or PBS at ZT13, relative clock mRNA levels (normalized to β-actin) were determined by qPCR 48 h after challenge in splenocytes, and calculated as percentage of the maximum value. Data are mean + SEM of 5 and 6 animals per time point for the control and poly(I:C) challenge group, respectively. ns, not significantly different from the control (PBS challenge) as per two-tailed t test.
(TIF)

S2 Fig Daily variations of mRNA levels in splenocytes. Daily variations in *Per2*, *Rev-erbα*, and *Tlr* gene expression in splenocytes. Relative mRNA levels at each time point were determined by qPCR and calculated as the relative expression over the 24-h period. Data are mean ± SEM of 5 animals per time point. One-way analysis of variance (ANOVA) was used to make comparisons between the acrophase and other time points. *p < 0.05, **p < 0.01, *** *p* < 0.001. Open bar indicates light period, while colored bar indicates dark period.
(TIF)

S1 File qPCR dataset. (XLSX)

S2 File qPCR raw dataset. (XLSX)
