# Supplementary figures and images for: Pathogen-associated molecular patterns alter molecular clock gene expression in mouse splenocytes
Source: PLoS One. 2017 Dec 18;12(12):e0189949. doi: 10.1371/journal.pone.0189949 (PMC5734770; doi:10.1371/journal.pone.0189949)

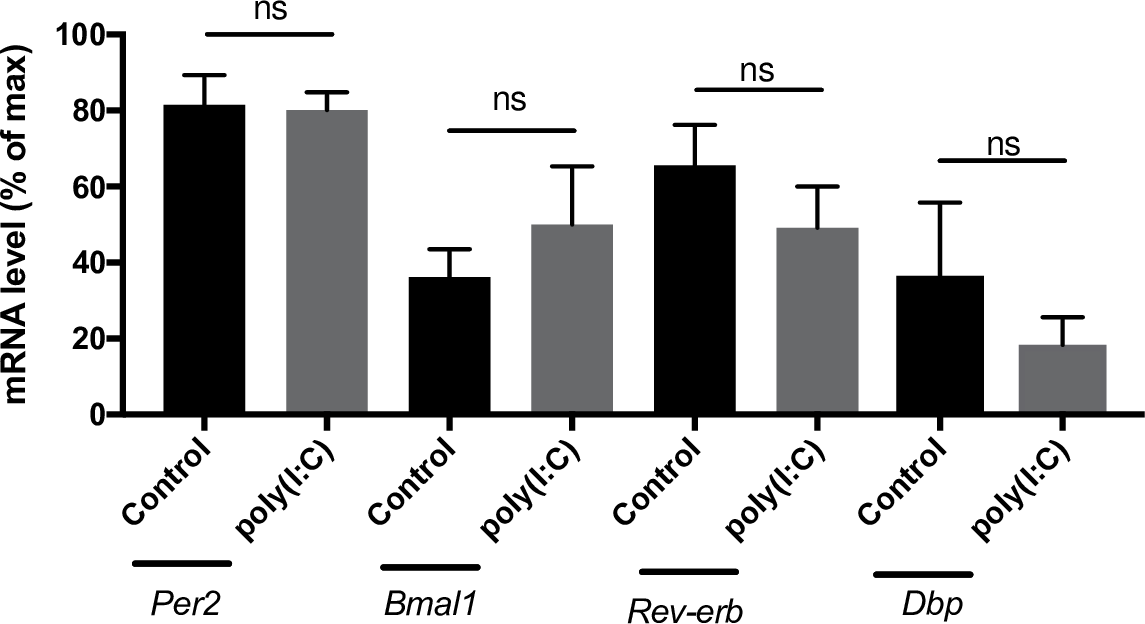

Supplement: S1 Fig — Mice were challenged with poly(I:C) or PBS at ZT13, relative clock mRNA levels (normalized to β-actin) were determined by qPCR 48 h after challenge in splenocytes, and calculated as percentage of the maximum value. Data are mean + SEM of 5 and 6 animals per time point for the control and poly(I:C) challenge group, respectively. ns, not significantly different from the control (PBS challenge) as per two-tailed t test. (TIF) [file pone.0189949.s001.tif]

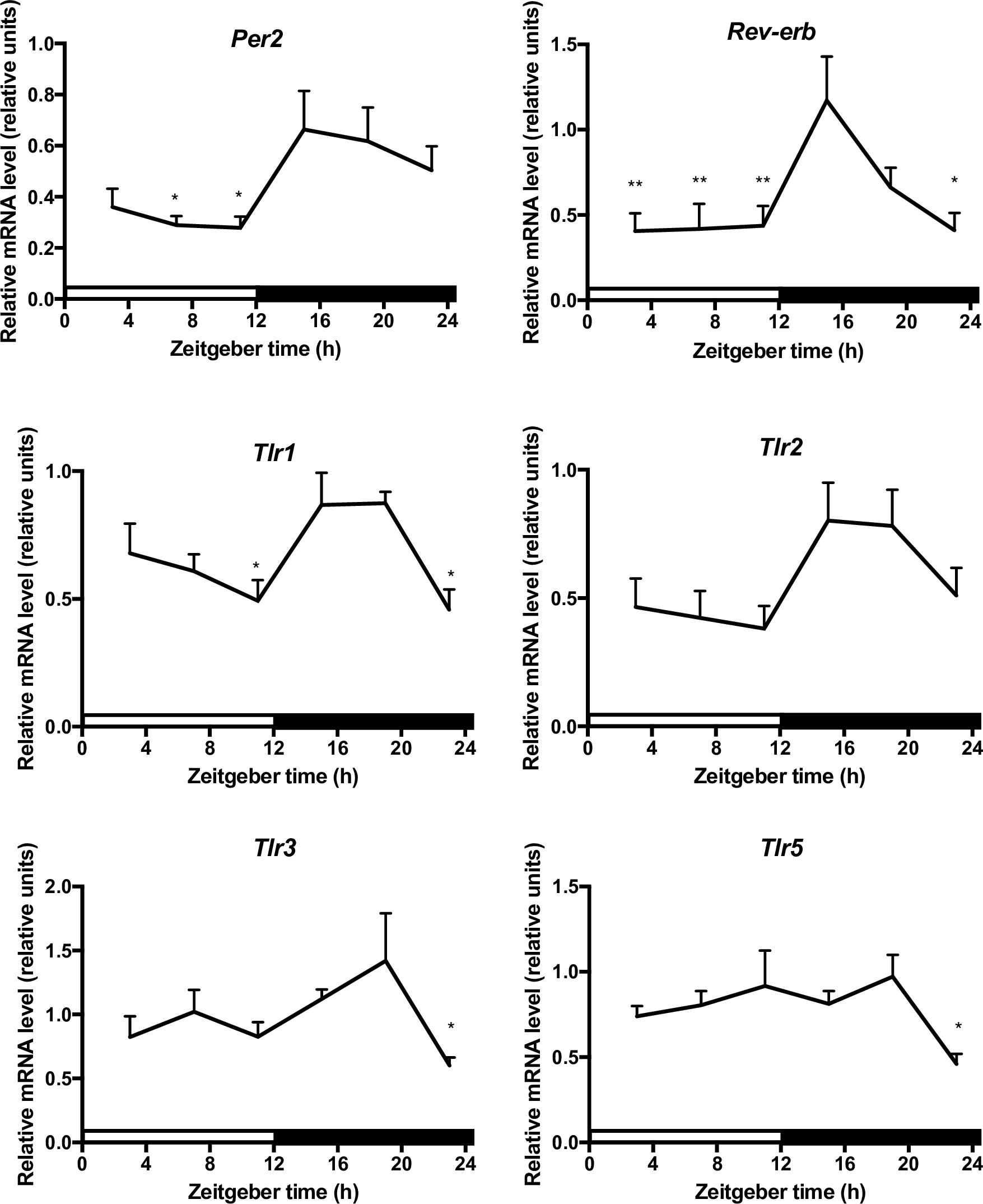

Supplement: S2 Fig — Daily variations in Per2, Rev-erbα, and Tlr gene expression in splenocytes. Relative mRNA levels at each time point were determined by qPCR and calculated as the relative expression over the 24-h period. Data are mean ± SEM of 5 animals per time point. One-way analysis of variance (ANOVA) was used to make comparisons between the acrophase and other time points. *p < 0.05, **p < 0.01, *** p < 0.001. Open bar indicates light period, while colored bar indicates dark period. (TIF) [file pone.0189949.s002.tif]
